# Classical Hodgkin lymphoma-type and monomorphic-type post-transplant lymphoproliferative disorder following liver transplantation: a case report

**DOI:** 10.1186/s40792-018-0480-x

**Published:** 2018-07-06

**Authors:** Hiroyuki Kumata, Chikashi Nakanishi, Keigo Murakami, Shigehito Miyagi, Noriko Fukuhara, Joaquim Carreras, Naoya Nakamura, Ryo Ichinohasama, Michiaki Unno, Takashi Kamei, Hironobu Sasano

**Affiliations:** 1 Department of Surgery, Graduate School of Medicine, Tohoku University, 1-1 Seiryou-machi, Aobaku, Sendai, 980-8574 Japan; 2 Department of Pathology, Graduate School of Medicine, Tohoku University, 1-1 Seiryou-machi, Aobaku, Sendai, Miyagi, 980-8574 Japan; 3 Department of Hematology and Rheumatology, Graduate School of Medicine, Tohoku University, 1-1 Seiryou-machi, Aobaku, Sendai, Miyagi, 980-8574 Japan; 4 Department of Pathology, School of Medicine, Tokai University, 143 Shimokasuya, Isehara, Kanagawa, 259-1193 Japan; 5 Division of Hematopathology, Tohoku University Hospital, 1-1 Seiryou-machi, Aobaku, Sendai, Miyagi, 980-8574 Japan

**Keywords:** Post-transplant lymphoproliferative disorder, Classical Hodgkin lymphoma, Liver transplantation

## Abstract

**Background:**

Post-transplant lymphoproliferative disorder (PTLD) is a life-threatening complication that can be difficult to treat; moreover, determination of the pathophysiological type is difficult. We report a rare case of a patient who developed two types of Epstein–Barr virus (EBV)-negative PTLD following living donor liver transplantation (LDLT).

**Case presentation:**

A 64-year-old man underwent LDLT for acute fulminant hepatitis B. Sixty-five months later, he developed EBV-negative monomorphic B cell PTLD. Reduction of immunosuppressive therapy and chemotherapy with rituximab resulted in a partial response. He received radioimmunotherapy with yttrium-90-ibritumomab tiuxetan, which was effective for all lesions, except for the splenic hilar lesion, which enlarged and seemed to penetrate the stomach. Therefore, he underwent resection of the pancreatic tail with splenectomy and partial gastrectomy. The pathological diagnosis was EBV-negative classical Hodgkin lymphoma (cHL)-type PTLD.

**Conclusions:**

This patient showed an unexpected course of PTLD, from both a clinical and pathological perspective. There are no prior reports of an adult case of EBV-negative cHL-type PTLD coexisting with EBV-negative monomorphic B cell PTLD. When a strange and refractory lesion persists despite effective therapy for PTLD, we must consider the possibility of another type of PTLD within the residual lesion.

## Background

Post-transplant lymphoproliferative disorder (PTLD) and susceptibility to infection are important and severe complications that occur secondary to the clinical use of potent immunosuppressive agents. According to 2008 World Health Organization classification, PTLD is categorized as one of the four major histological forms: early lesion, polymorphic PTLD, monomorphic PTLD, and classical Hodgkin lymphoma-type PTLD (cHL). In the clinic settings, monomorphic PTLD is observed most frequently, whereas cHL-type PTLD is uncommon [1]. It is suggested that most PTLD cases have a strong relationship with Epstein–Barr virus (EBV) [2]. Here we report a rare case of EBV-negative cHL-type PTLD, coexisting with EBV-negative monomorphic B cell PTLD following living donor liver transplantation (LDLT).

## Case presentation

**Fig. 1:**
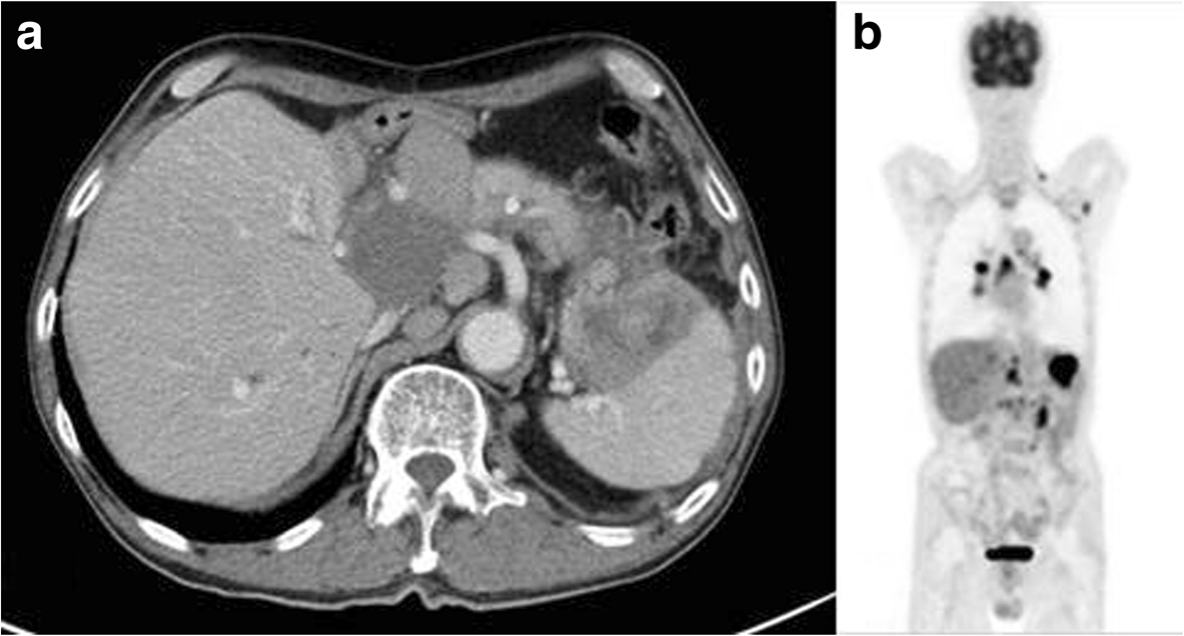
Imaging findings at the time of admission. **a** The first computed tomography (CT) demonstrated systemic lymphadenopathy, mainly in the abdomen. The large lymphadenopathy in the splenic hilum seemed to be a gastric submucosal tumor. **b** Fluorodeoxyglucose positron emission tomography showed systemic uptake, mainly in the intraabdominal lymph nodes. The largest uptake corresponded to the lymphadenopathy in the splenic hilum, as observed on CT

A 64-year-old man underwent LDLT from his daughter in May 2009 for acute fulminant hepatitis B. Both the recipient and donor had prior infection with EBV. The initial immunosuppression consisted of methylprednisolone and tacrolimus, with induction therapy using basiliximab. The trough level of tacrolimus was adjusted within the range of 3–4 ng/ml. Thereafter, he received tacrolimus (3 mg/day) and mycophenolate mofetil (500 mg/day), which kept the graft function in good condition. He had no history of immunological rejection in post-operative course until 65 months following LDLT, when he noted fever, pain in the left epigastrium, and nausea. He underwent computed tomography (CT) as a follow-up just 1 year before the onset of this symptom, but no abnormal findings were found in particular. CT revealed systemic lymphadenopathy, mainly in the abdomen, mediastinum, and bilateral cervical lymph nodes. In the splenic hilum, there was a large lymphadenopathy that compressed the stomach (Fig. 1a).

**Fig. 2:**
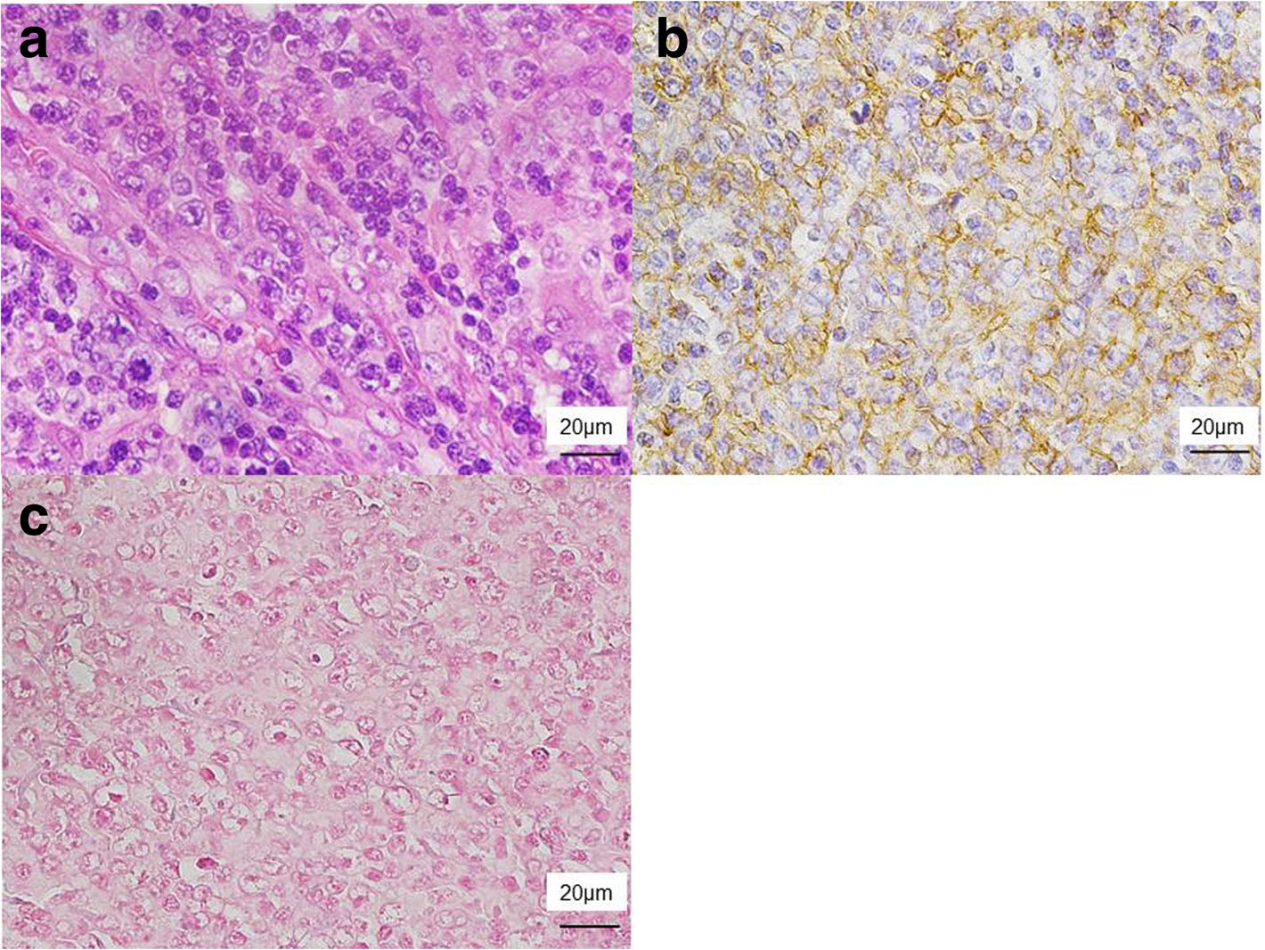
Microscopic findings of biopsy from the cervical lymph node. **a** Hyperplasia of large and pleomorphic atypical lymphoid cells was observed in the lymph nodes (hematoxylin/eosin). **b** Immunohistochemical staining was positive for CD20. **c** Epstein–Barr virus-encoded ribonucleic acid in situ hybridization was negative in the tumor cells

Upper gastrointestinal endoscopy revealed that a part of the gastric wall was compressed by the large lymphadenopathy in the splenic hilum on CT. We performed a biopsy from the lesion of the stomach; however, the result was inflammatory mucosa only, and we could not find a definitive diagnosis. Fluorodeoxyglucose positron emission tomography (FDG-PET) also showed systemic uptake corresponding to the area of lymphadenopathy on CT (Fig. 1b). His EBV viral load in the blood was undetectable. Biopsy from the cervical lymph node showed diffuse distortion of architecture, with hyperplasia of large and pleomorphic atypical lymphoid cells (Fig. 2a).

**Fig. 3:**
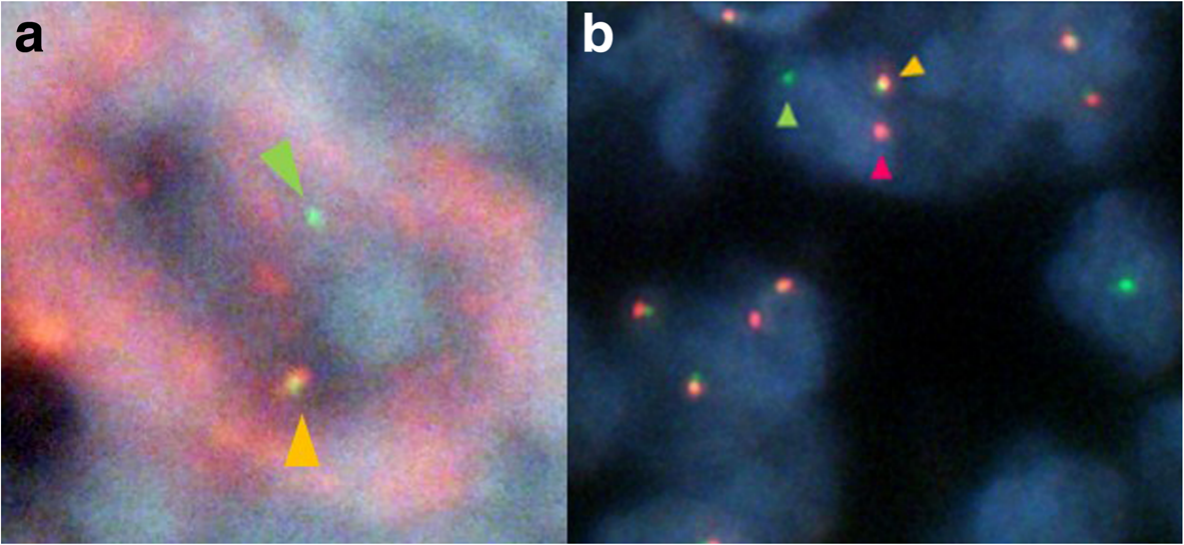
Fluorescence in situ hybridization (FISH) findings of each post-transplant lymphoproliferative disorder (PTLD). **a** FISH of monomorphic B cell (follicular lymphoma type) PTLD. The split signals (green and yellow triangles) indicating a rearrangement of BCL6 in the tumor cell. **b** Double labeling detection method that combines FISH for rearrangement of BCL6 and immunohistochemical staining for CD30 of classical Hodgkin lymphoma-type PTLD. In the Hodgkin cells, there were the split signals (green and yellow triangles) indicating a rearrangement of BCL6, and the other signal (red triangle) indicating an immunohistochemical positivity for CD30

Flow cytometry for abnormal B cell populations revealed the following phenotypes: CD20+, CD10+, CD3−, CD56−, CD4−, and CD30−. Antibodies used for immunohistochemistry showed CD20+, CD10+, CD3−, CD5−, CD45+, CD56−, CD79a+, bcl2−, and bcl6+ (weak) (Fig. 2b and Table 1). EBV-encoded ribonucleic acid in situ hybridization (EBER-ISH) was negative in the tumor cells (Fig. 2c). Chromosome analysis demonstrated 47,X,−Y,+X,add(3)(q27),+ 5,del(6)(p23),add(10)(q26). Polymerase chain reaction analysis showed rearrangement of the IgH gene. The histopathological diagnosis was follicular lymphoma (FL) grade 3B, with split signals of BCL6 gene defined by fluorescence in situ hybridization (FISH) (Fig. 3a). From these findings, he was diagnosed with EBV-negative monomorphic B-cell PTLD.

**Table 1:** Immunohistochemical findings of each post-transplant lymphoproliferative disorder (PTLD)

Antibody	1st PTLD	2nd PTLD
CD3	–	–
CD5	–	–
CD10	+	+
CD20	+	–
CD30	None	+
CD45	+	+
CD56	–	–
CD79a	+	+

*1st PTLD* Epstein–Barr virus (EBV)-negative monomorphic B-cell PTLD, *2nd PTLD* EBV-negative classical Hodgkin lymphoma-type PTLD

**Fig. 4:**
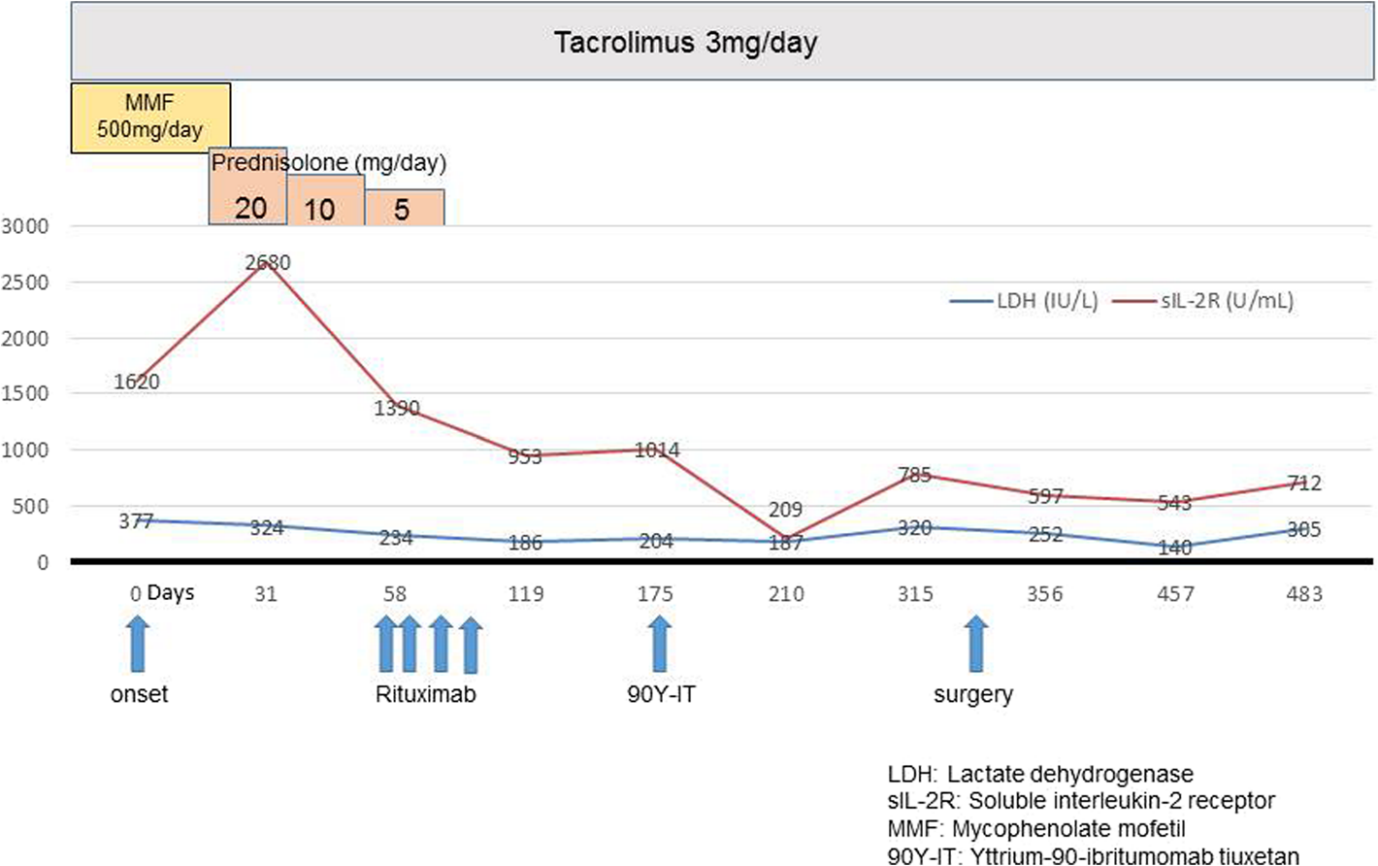
Clinical course

**Fig. 5:**
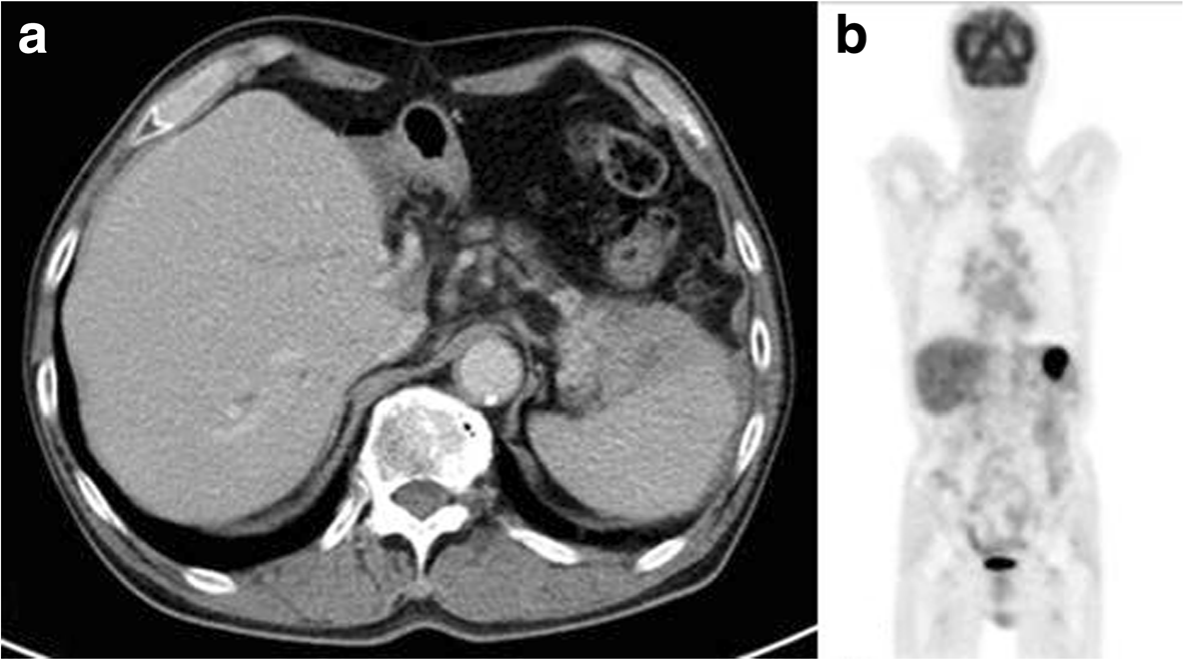
Preoperative image findings. **a** Preoperative computerized tomography and **b** fluorodeoxyglucose positron emission tomography demonstrated almost complete resolution of the systemic lymphadenopathy, except for the splenic hilar lymph node

The clinical course with the progress of serum lactate dehydrogenase and soluble interleukin-2 receptor is shown in Fig. 4. His immunosuppressive therapy was reduced. His target trough level of tacrolimus was adjusted to ≤ 4 ng/ml at the onset of PTLD, which is the target trough level during the treatment of PTLD at our institution. Therefore, we elected to discontinue mycophenolate mofetil without changing the dose of tacrolimus. Moreover, he received prednisolone, but there was minimal response. Thereafter, he received rituximab therapy; unfortunately, this treatment was slightly effective and the splenic hilar lymph node remained unchanged in size. Subsequently, he received radioimmunotherapy with yttrium-90-ibritumomab tiuxetan (90Y-IT). The agent 90Y-IT contains the anti-CD20 monoclonal antibody ibritumomab covalently bonded to the chelating agent tiuxetan and radiolabeled with 90Y. Three months after the therapy, the systemic lymphadenopathy resolved almost completely, except for the splenic hilar lesion, which seemed to progress uncontrollably and penetrate the stomach (Fig. 5a, b).

**Fig. 6:**
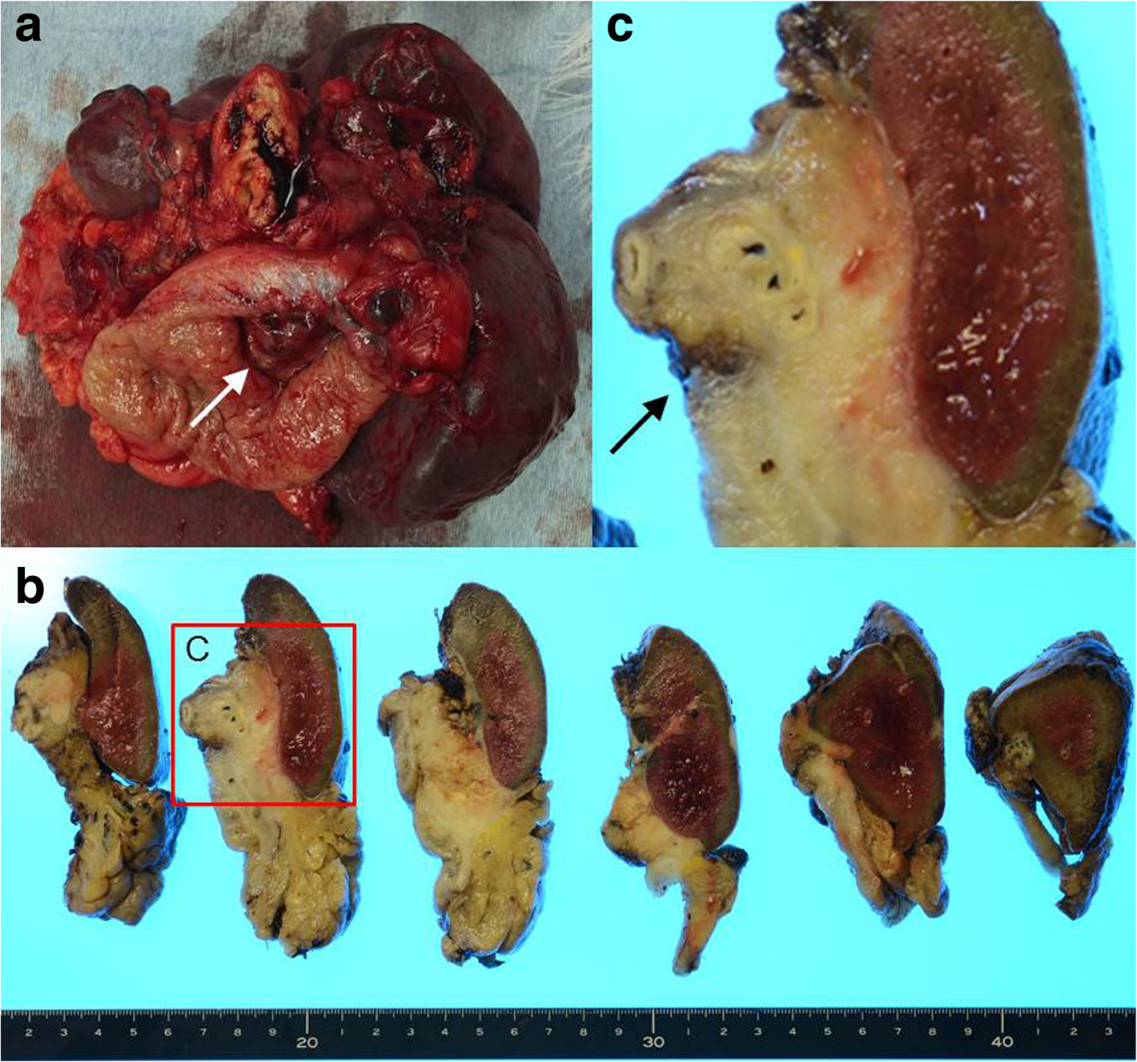
Macroscopic findings of the excised specimen. **a** A mass was exposed in the lumen of the stomach (white arrow). **b**, **c** The splenic hilar lesion was enlarged to compress the surroundings, penetrating the stomach (black arrow)

**Fig. 7:**
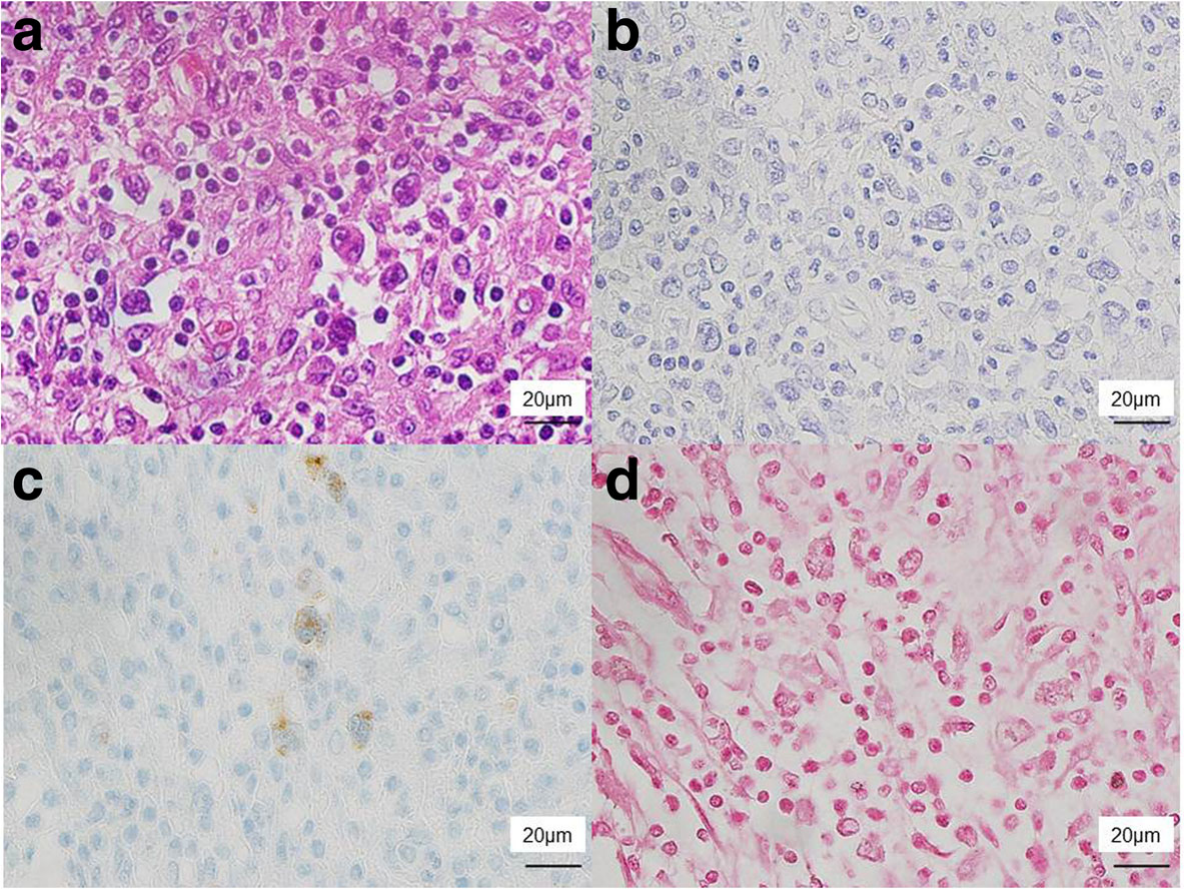
Microscopic findings of the excised specimen. **a** A few large dyskaryotic or multinucleated atypical lymphoid cells, including Reed–Sternberg cells, were observed (hematoxylin/eosin). Immunohistochemical staining revealed that they were negative for **b** CD20 and positive for **c** CD30. The results of other immunostaining were described in Table 1. **d** Epstein–Barr virus-encoded ribonucleic acid in situ hybridization was negative in the tumor cells

The patient underwent resection of the pancreatic tail with splenectomy and partial gastrectomy (Fig. 6a–c). The splenic hilar lesion was exposed in the lumen of the stomach (Fig. 6a). It was enlarged and compressed its surroundings, penetrating the stomach (Fig. 6b, c). Morphologically, the specimen revealed few large dyskaryotic or multinucleated atypical lymphoid cells infiltrating the stomach wall, pancreas, and splenic artery and vein (Fig. 7a). The atypical lymphoid cells were suggested to be Hodgkin/Reed–Sternberg cells. Antibodies used for immunohistochemistry showed CD20−, CD3−, CD5−, CD45+, CD56−, and CD79a+ and weak positivities for both CD30 and CD10 (Fig. 7b, c and Table 1). To reveal the relationship between the Hodgkin cells and the underlying monomorphic PTLD, we performed a double labeling detection method that combined FISH for breakpoint in BCL6 and immunohistochemical staining for CD30 on the Hodgkin cells, revealing rearrangement of BCL6 (Fig. 3b). EBER-ISH showed negativity in the tumor cells (Fig. 7d). His preoperative EBV viral load in the blood was also undetectable. Therefore, we diagnosed EBV-negative cHL-type PTLD.

Six months after the surgery, surveillance CT showed no recurrence of PTLD; the patient then resumed tacrolimus (3 mg/day).

### Discussion

PTLD is regarded as distinct from hematological malignancies, which develop in non-transplant recipients [1]. PTLD has various origins; approximately 85% are B cell proliferations, 14% are T cell proliferations, and the remaining 1% are NK cell or plasmacyte proliferations [3].

The main risk factors for PTLD are age, EBV status, use of immunosuppressive agents, and the type of organ transplanted [2]. Most adults with PTLD are EBV-negative, whereas children with PTLD tend to be EBV-positive [1]. As a patient receives higher doses of immunosuppressive agents, the frequency of developing PTLD increases and the time to onset of PTLD shortens [4]. In our patient, therapeutic drug monitoring kept the levels of immunosuppressive agents in the appropriate range. There is a difference in the prevalence of PTLD according to the type of organ that is transplanted; the small intestine is the most affected (20%), followed by the lungs at 4–10%, heart at 1–6%, liver and kidneys at 1–3%, and other organs at an average of 10% [5]. At our institution, we encountered 95 pediatric cases (< 18 years old) and 75 adult cases who underwent LDLT between July 1991 and December 2017. Among these, seven pediatric patients (7%) and two adult cases (3%) developed PTLD.

PTLD has a strong relationship with EBV, particularly in B cell proliferations [2]. Krasuska-Sławińska et al. reported that EBV has been identified in 90% of B cell-proliferating PTLDs [6]. In general, the time of development of EBV-negative PTLD occurs later than that of EBV-positive PTLD [7]. These features were seen in our case. EBV-negative PTLD remains lesser elucidated than EBV-positive PTLD. Among the nine PTLD cases that we have encountered at our institution, all seven pediatric PTLDs were EBV-positive, whereas both adult cases were EBV-negative. In general, the first choice of treatment for PTLD is the reduction of immunosuppression. Rituximab is theoretically effective for CD20-positive PTLD but is only effective in approximately half of these patients [8]. In our case, considering that this patient was older and that the type of PTLD would be enough response by steroids, we did not initially use rituximab. However, because it was not possible to attain the expected response, we used rituximab. Recently, 90Y-IT has been reported to be effective in PTLD patients who were resistant to rituximab [8] as well as in patients with CD20-positive cHL [9]. In our case, 90Y-IT seemed to be effective for monomorphic PTLD but ineffective for cHL-type PTLD because of the negativity to CD20. Surgical therapy can also be effective and provide the correct diagnosis for localized PTLD, like in our cases [10]. His target trough of tacrolimus has been adjusted at a low level of ≤4 ng/ml both before and during PTLD [11]. Moreover, because he did not show recurrence and rejection findings after surgery, there was no need to change the target trough, and thus, he was observed at the same dose of tacrolimus. For data pertaining to adjuvant chemotherapy, we searched PubMed and found no studies that reported on using adjuvant chemotherapy for PTLD after curative surgery. Further cases and studies are needed to reveal the effectiveness of adjuvant chemotherapy for PTLD after surgery.

Our case exhibited various histopathological types of PTLD. It was difficult to determine whether the cHL-type PTLD developed after the remission of monomorphic B cell PTLD; the two types of PTLD might have coexisted from the beginning. There are reports of two pediatric liver transplant patients [12–14] and one adult patient [15] who developed cHL-type PTLD subsequent to another type of PTLD (Table 2); all cases developed EBV-positive cHL-type PTLD after EBV-positive polymorphic B-cell PTLD [12, 14, 15]. Our case was very interesting due to simultaneous presentation of EBV-negative cHL-type PTLD and EBV-negative monomorphic B-cell PTLD.

**Table 2:** Features of classical Hodgkin lymphoma (cHL)-type post-transplant lymphoproliferative disorder (PTLD) subsequent to another PTLD following liver transplantation

Case no.	Age/sex	Interval	Diagnosis	EBV positivity	Symptoms except lymphoadenopathy	Treatment	Outcome	Reference
1	64 years/M	5 years and 5 months from transplant	monomorphic PTLD	−	Fever, abdominal pain	Reduction of immunosupression, rituximab, 90Y-IT	*	Current case
		1 year from the 1st PTLD	cHL PTLD	−	Fever	Surgery	CR	
2	28 years/F	2 years and 2 months from transplant	Polymorphic PTLD	+	Fever	Reduction of immunosupression, acyclovir	CR	15
		2 years and 5 months from the 1st PTLD	cHL PTLD	+	Fever, splenomegaly	Reduction of immunosupression, acyclovir, MOPP, splenectomy	CR	
3	9 years/M	8 years from transplant	Polymorphic PTLD	+	Enlarged tonsil, adenoid	Reduction of immunosupression, acyclovir	CR	12
		6 years and 10 months from the 1st PTLD	cHL PTLD	+	Mediastinal mass	COPP, ABV	CR	
4	6 years/M	4 years and 2 months from transplant	Polymorphic PTLD	+	None	Reduction of immunosupression, gancyclovir	CR	14
		1 year and 6 months from the 1st PTLD	cHL PTLD	+	Fever	MOPP, ABVD, radiation	CR	

*PTLD* post-transplant lymphoproliferative disorder, *cHL* classical Hodgkin lymphoma, *M* male, *F* female, *90Y-IT* yttrium-90-ibritumomab tiuxetan, *MOPP* mechlorethamine, vincristine, procarbazine, prednisone, *COPP* cyclophosphamide, vincristin, procarbazine, prednisone, *ABV* doxorubicin, bleomycin, vinblastine, *ABVD* doxorubicin, bleomycin, vinblastine, dacarbazine, *CR* complete remission

*The systemic lymphadenopathy resolved almost completely, except for the splenic hilar lesion composed of cHL-type PTLD

In our case, the monomorphic PTLD showed rearrangement of BCL6 by FISH (Fig. 3a), whereas the cHL-type PTLD showed rearrangement of BCL6 during the double labeling detection method that combined FISH and immunohistochemical staining (Fig. 3b). From these results, we concluded that these two PTLDs might have a common origin. Several reports have shown that cHL and FL are derived from a shared germinal center B cell clone [16]. Nakamura et al. reported that FL converted to cHL by proving a rearrangement of BCL2 [17]. A rearrangement of BCL6 is mainly observed in FL or diffuse large B cell lymphoma and is rarely observed in cHL. With regard to PTLD cases, Poirel et al. reported three cases of monomorphic type and one case of cHL type with rearrangement of BCL6 [18]. Five percent of patients who developed EBV-related PTLD developed another EBV-related PTLD, but there are no reports that can prove a causal relationship between the two types of PTLD [19]. To the best of our knowledge, there has been no report of EBV-negative monomorphic B cell PTLD converting to EBV-negative cHL-type PTLD by a rearrangement of BCL6 in the same patient following SOT.

## Conclusions

When a strange and refractory PTLD lesion persists despite effective therapy, practitioners should consider the possibility of another type of PTLD in the residual lesion.

## Abbreviations

PTLD: Post-transplant lymphoproliferative disorder
cHL: Classical Hodgkin lymphoma
EBV: Epstein–Barr virus
LDLT: Living donor liver transplantation
CT: Computerized tomography
FDG-PET: Fluorodeoxyglucose positron emission tomography
EBER-ISH: Epstein–Barr virus-encoded ribonucleic acid in situ hybridization
FL: Follicular lymphoma
FISH: Fluorescence in situ hybridization
90Y-IT: Yttrium-90-ibritumomab tiuxetan
